# Varporter: a software platform for comprehensive genomic profiling (CGP) in cancer

**DOI:** 10.1038/s41439-026-00357-z

**Published:** 2026-07-24

**Authors:** Masashi Idogawa, Kohichi Takada, Tasuku Mariya, Aki Ishikawa, Satoshi Iyama, Tsuyoshi Saito, Hiroshi Nakase, Masayoshi Kobune, Akihiro Sakurai

**Affiliations:** 1 Division of Medical Genome Sciences, Department of Genomic and Preventive Medicine, Sapporo Medical University School of Medicine, Sapporo, Japan; 2 Division of Gastroenterology and Hepatology, Department of Internal Medicine, Sapporo Medical University School of Medicine, Sapporo, Japan; 3 Division of Medical Oncology, Department of Internal Medicine, Sapporo Medical University School of Medicine, Sapporo, Japan; 4 Division of Clinical Genomics, Department of Genomic and Preventive Medicine, Sapporo Medical University School of Medicine, Sapporo, Japan; 5 Department of Obstetrics and Gynecology, Sapporo Medical University School of Medicine, Sapporo, Japan; 6 Division of Hematology, Department of Internal Medicine, Sapporo Medical University School of Medicine, Sapporo, Japan

**Keywords:** Computational biology and bioinformatics, Cancer genomics, Genomic analysis

## Abstract

The use of comprehensive genomic profiling (CGP) in cancer, also known as cancer gene panel tests, continues to expand; however, the interpretation of the results of these tests requires a tremendous amount of manual work. To improve the efficiency of this evaluation process, we developed the CGP support software named Varporter. This software automatically imports multiple data files and can display various information contained within the CGP results. In tumor-only tests, *TP53* variants often require pathogenicity evaluation as presumed germline pathogenic variants. Varporter enables the automated determination of several criteria in the Variant Interpretation Guidelines established by the ClinGen *TP53* Variant Curation Expert Panel. Direct links to the websites on therapeutic evidence are also automatically generated from each variant, and multiple test reports can be summarized onto a single sheet, thus making it easy to review newly added variants to clinical trials. Varporter has made it possible to automate and streamline complicated manual tasks related to CGP evaluation and to redirect human resources to more essential tasks. Varporter is designed for use with two CGP tests covered by Japanese national health insurance, FoundationOne CDx and Guardant360 CDx.

As various molecularly targeted therapies are being developed, the identification of the genetic mutations that are present in cancer is becoming essential for determining the optimal treatment approach. Since June 2019, five comprehensive genomic profiling (CGP) methods using next-generation sequencers in solid tumors have been covered by public insurance in Japan. These include three tissue-based methods (FoundationOne^®^ CDx, Oncoguide^TM^ NCC Oncopanel System, and GenMineTOP^®^ Cancer Genomic Profiling analysis) and two liquid biopsy methods (FoundationOne Liquid CDx and Guardant360^®^ CDx). Clinicians select among these options on the basis of factors such as specimen type, test characteristics, and clinical context. In March 2025, the first CGP method for hematological malignancies, HemeSight^®^, was covered by public insurance and is expected to be effective for diagnosis and treatment. By March 2025, CGP testing had been performed on more than 100,000 patients, with more than 2000 new cases being diagnosed each month and more than 20,000 cases per year expected to continue to be diagnosed.

The use of CGP in cancer (also known as cancer gene panel tests) continues to expand; however, the interpretation of the results of these tests requires a tremendous amount of manual work. One reason for this scenario involves the lack of software that is capable of properly reading the data files in formats such as XML or JSON, which contain text with low readability within the test results. Consequently, the human resources that are necessary for medical institutions to properly evaluate test results are limited in reality. To improve the efficiency of this evaluation process, we developed the CGP support software named Varporter. This software operates within Excel, automatically imports multiple data files, and can display various information contained within the CGP results. Varporter is not intended to provide automated clinical interpretation or classification; rather, it aims to assist clinicians and other related professionals by integrating annotation sources and automating reproducible processes in accordance with expert panel guidelines.

In cancer, CGP can detect not only somatic mutations for drug target discovery but also pathogenic germline variants, known as secondary findings (SFs). With respect to SFs, from the perspective of the patient’s psychological burden, the disclosure of only those findings that are beneficial for disease prevention, early detection, and treatment is desirable. In Japan, in addition to the ACMG’s SF list^1^, the disclosure recommendation list developed by a Japanese research group is used as a criterion for this scenario. However, most CGP results do not include this information. Therefore, Varporter has enabled the display of disclosure recommendation levels using these criteria.

The CGP methods involved in cancer are divided into two types: tumor-only tests, such as FoundationOne CDx (which analyzes only the tumor), and matched-pair tests (which analyze both tumor and non-tumor tissues). In the case of the former, distinguishing somatic mutations from germline variants is difficult, thereby necessitating evaluation as presumed germline pathogenic variants. The *TP53* gene is known to frequently undergo somatic mutations, whereas germline variants cause Li–Fraumeni syndrome, which leads to various hereditary tumors. Consequently, the evaluation of the pathogenicity of *TP53* variants as presumed germline pathogenic variants is often needed. However, the assessment of each criterion in the Variant Interpretation Guidelines established by the ClinGen *TP53* Variant Curation Expert Panel (VCEP) is highly complicated^2^. Varporter enables the automated determination of several ACMG criteria based on the guidelines.

## Various features of Varporter

### Display of sample information

**Fig. 1:**
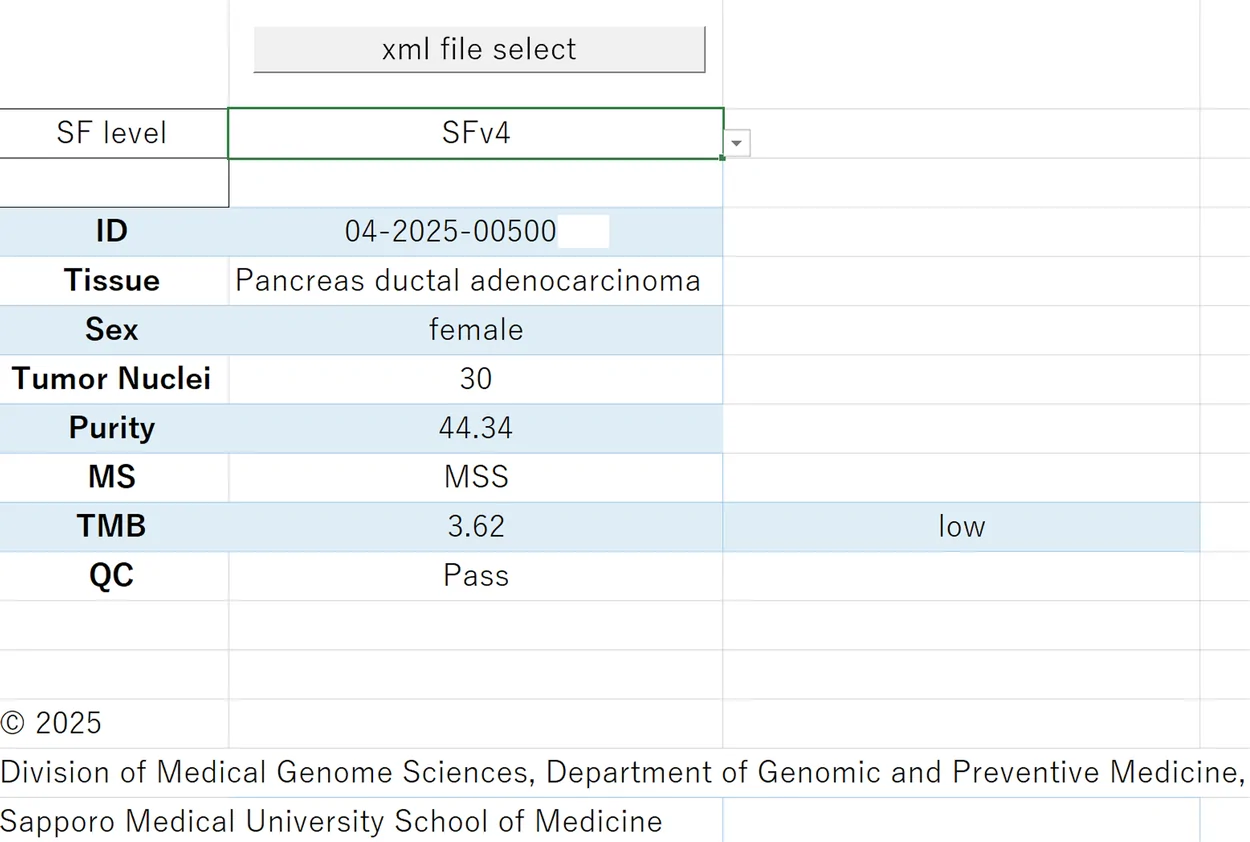
Various sample information displayed by Varporter. Case ID, tissue name, sex, proportion of tumor nuclei, purity, microsatellite instability (MSI) status (MSI-H, microsatellite instability-high; MSS, microsatellite stable), tumor mutational burden (TMB) score, and quality control (QC) metrics are extracted directly from the CGP result XML file and displayed. The SF level column indicates the secondary findings (SFs) disclosure recommendation grade applicable to each variant based on the selected guideline version: SFv3 and SFv4 refer to the Secondary Findings Disclosure Recommendation List Ver3.1 (2021) and Ver4.2 (2023) developed under a Japanese Ministry of Health, Labour, and Welfare research grant; and 2025 refers to the GPV/PGPV Management Guidelines 2025 Edition (https://www.iden-gan.jp/pgpv).

To display information, the [xml file select] button is pressed to select an XML file. The files are automatically imported into Excel, and case ID, tissue name, sex, proportion of tumor nuclei, purity, microsatellite instability score, tumor mutational burden score, and quality control information are displayed (Fig. 1). For Guardant360 CDx, whose results are provided as an Excel file, the corresponding file is selected in the same way, and the sample and variant information are imported and displayed in the same manner.

### Summary of single-nucleotide variations and copy number variations

**Fig. 2:**
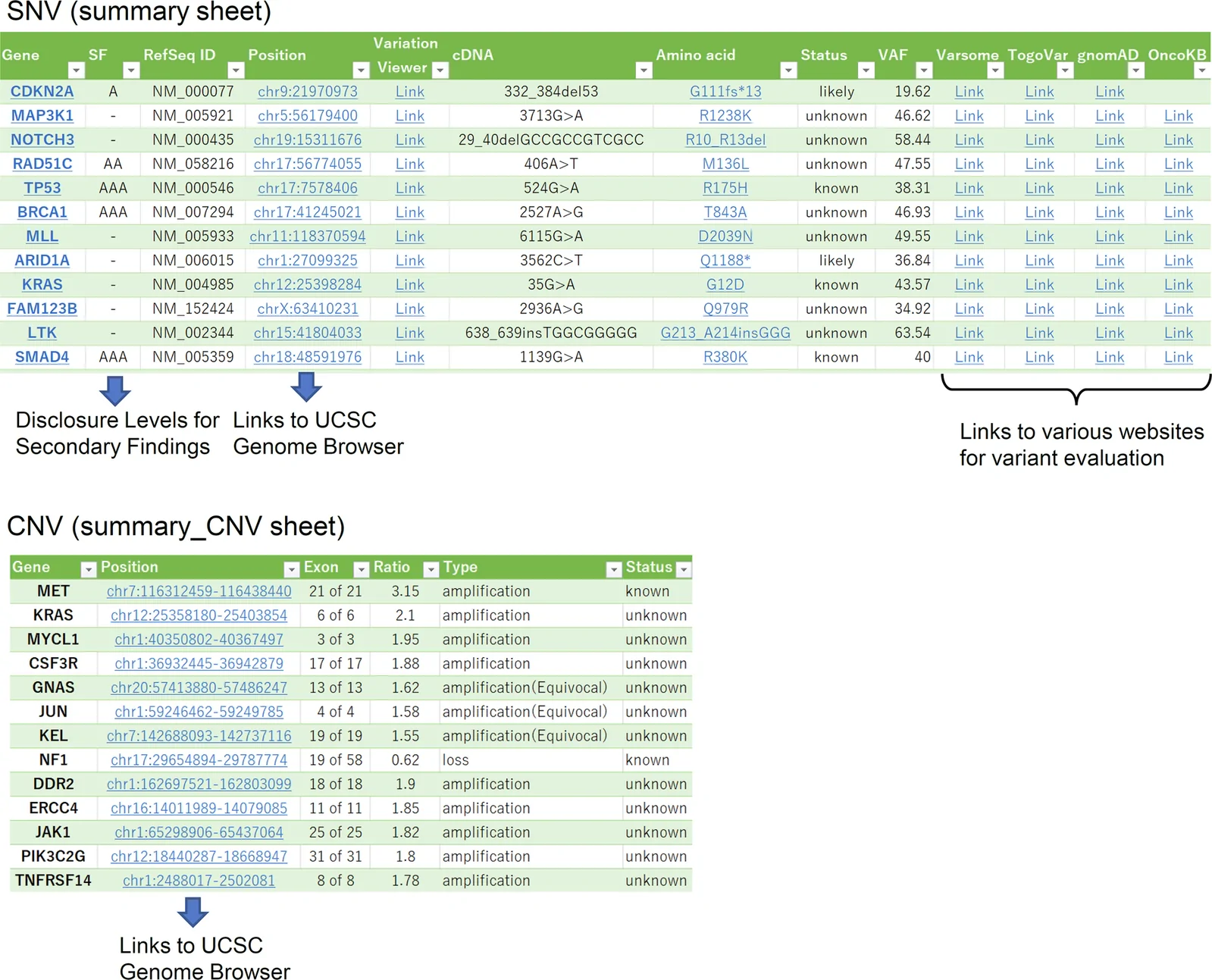
Integrated information table for SNVs and CNVs in Varporter. The secondary finding (SF) column displays the disclosure recommendation grade for each variant based on Japan-specific secondary findings guidelines (SFv3/SFv4: grades AAA, AA, A, B; 2025 edition: grades A and B), in which grades A and above represent the primary disclosure-recommended genes in clinical practice. Hyperlinks to VarSome, TogoVar, gnomAD, OncoKB, and CADD are automatically generated for each variant to facilitate access to external annotation resources. Copy number variations (CNVs) are displayed on a separate CNV summary sheet (lower table), showing the gene, genomic position, affected exons, copy number ratio, alteration type, and status, with a link to the UCSC Genome Browser provided for each entry. cDNA, complementary DNA; SNV, single-nucleotide variation; VAF, variant allele frequency.

In the single-nucleotide variation (SNV) sheet, the gene name, genomic position, complementary DNA and amino acid changes, and variant allele frequency are displayed for each variant in a table (Fig. 2). Links to various sites useful for variant assessment based on nucleotide or amino acid changes are also automatically generated (Fig. 2, right side). These sites include the UCSC Genome Browser (genomic information), gnomAD and TogoVar (allele frequencies in the general population), OncoKB (therapeutic evidence), VarSome (variant evaluation), and CADD (variant pathogenicity scores). By clicking on these links, users are able to directly access the search results page for each site. It is also possible to import Center for Cancer Genomics and Advanced Therapeutics (C-CAT) reports (which include information on genotype-matched clinical trials) into Excel sheets, where the evidence level is displayed; moreover, links from each SNV are generated to the relevant sections.

In addition to SNVs, the copy number variation (CNV) data contained in the XML file are displayed on a dedicated CNV summary sheet (Fig. 2). For each CNV, the gene name, genomic position, affected exons, copy number ratio, alteration type (for example, amplification or loss), and status are extracted from the XML and displayed. A link to the UCSC Genome Browser is provided for each CNV.

### Display of disclosure recommendation levels for SFs

On the basis of the disclosure recommendation criteria for SFs selected in the filter sheet, the corresponding disclosure level for the gene is displayed (Fig. 2, second column). Variants can be filtered and extracted based on this level.

### ACMG criteria determination based on ClinGen *TP53* VCEP

**Fig. 3:**
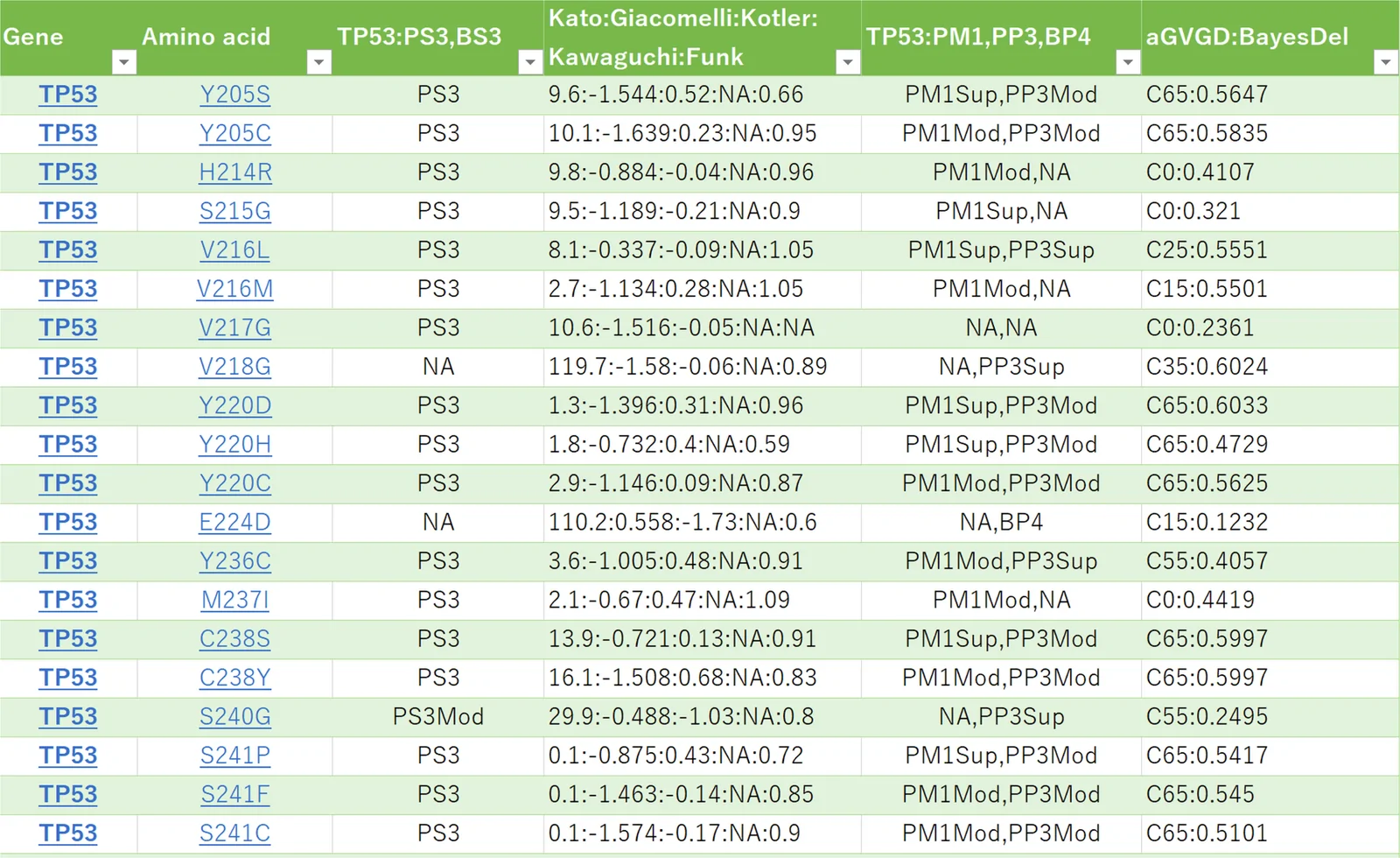
Output of pathogenicity assessments for *TP53* variants in Varporter. PS3, BS3, PM1, PP3, and BP4 are automatically determined according to the ClinGen TP53 Expert Panel Specifications, according to the ACMG/AMP Variant Interpretation Guidelines for TP53 Version 2.4.0. The functional assay score columns displayed are: Kato (transactivation activity in yeast)^3^, Giacomelli (growth suppression assay in A549 cells)^4^, Kawaguchi (oligomerization assay in yeast, applicable to the tetramerization domain)^5^, Kotler (relative fitness scores in H1299 cells, applicable to the DNA binding domain)^6^, and Funk (CRISPR-based fitness scores)^7^. These assays are formally incorporated into the PS3/BS3 criteria of the ClinGen TP53 VCEP specifications.

In the SNV sheet, the following ACMG criteria for *TP53* variants are automatically assessed and displayed: PS3 and BS3 (which are based on well-established in vitro or in vivo functional studies^3–7^); PM1 (which is related to mutational hotspots); and PP3 and BP4 (which are based on in silico scores^8,9^) (Fig. 3).

With respect to the benefits offered by Varporter, the foremost advantage involves a substantial reduction in the time required for evaluation. Second, the elimination of simple tasks such as copy-pasting and subsequent searching reduces the mental burden on the evaluator by reducing the risk of making errors. Furthermore, the ability to refer to the results of various related databases by simply clicking on the links has an educational effect on the evaluators. Analysis results from multiple test reports can be summarized on a single sheet, thereby making it easy to create statistics and summaries within the facility and to review newly added variants to clinical trials. The Varporter system has made it possible to automate and streamline complicated manual tasks related to CGP evaluation, as well as to redirect human resources to more essential tasks. The number of tests is expected to increase in the future; moreover, the automation and streamlining of tasks using software are considered to be essential tasks for appropriate CGP evaluation. In the future, we plan to advance development to apply it not only to CGP but also to whole-exome and whole-genome sequencing analysis.

## Software availability

The basic version of the Varporter software can be downloaded from the following website (https://web.sapmed.ac.jp/canmol/varporter/basic.html) and is freely available for use.
